# Maxillary Bone Regeneration Based on Nanoreservoirs Functionalized *ε*-Polycaprolactone Biomembranes in a Mouse Model of Jaw Bone Lesion

**DOI:** 10.1155/2018/7380389

**Published:** 2018-02-26

**Authors:** Marion Strub, Xavier Van Bellinghen, Florence Fioretti, Fabien Bornert, Nadia Benkirane-Jessel, Ysia Idoux-Gillet, Sabine Kuchler-Bopp, François Clauss

**Affiliations:** ^1^INSERM, UMR 1260, Regenerative Nanomedicine (RNM), FMTS, 67000 Strasbourg, France; ^2^Faculté de Chirurgie-Dentaire, Université de Strasbourg, 8 rue Sainte Elisabeth, 67000 Strasbourg, France; ^3^Hôpitaux Universitaires de Strasbourg (HUS), Pôle de Médecine et Chirurgie Bucco-Dentaires, 1 place de l'Hôpital, 67000 Strasbourg, France

## Abstract

Current approaches of regenerative therapies constitute strategies for bone tissue reparation and engineering, especially in the context of genetical diseases with skeletal defects. Bone regeneration using electrospun nanofibers' implant has the following objectives: bone neoformation induction with rapid healing, reduced postoperative complications, and improvement of bone tissue quality.* In vivo* implantation of polycaprolactone (PCL) biomembrane functionalized with BMP-2/Ibuprofen in mouse maxillary defects was followed by bone neoformation kinetics evaluation using microcomputed tomography. Wild-Type (WT) and Tabby (Ta) mice were used to compare effects on a normal phenotype and on a mutant model of ectodermal dysplasia (ED). After 21 days, no effect on bone neoformation was observed in Ta treated lesion (4% neoformation compared to 13% in the control lesion). Between the 21st and the 30th days, the use of biomembrane functionalized with BMP-2/Ibuprofen in maxillary bone lesions allowed a significant increase in bone neoformation peaks (resp., +8% in mutant Ta and +13% in WT). Histological analyses revealed a neoformed bone with regular trabecular structure, areas of mineralized bone inside the membrane, and an improved neovascularization in the treated lesion with bifunctionalized membrane. In conclusion, PCL functionalized biomembrane promoted bone neoformation, this effect being modulated by the Ta bone phenotype responsible for an alteration of bone response.

## 1. Introduction

Approaches of bioengineering and regenerative medicine aim to create different types of materials, implants, or scaffold mimicking structure of extracellular matrix, functionalized with bioactive molecules or living cells. The clinical purpose of these methods is the reparation or guided regeneration of damaged tissue, in our case, jaw bone affected by genetical diseases. These biomembranes or scaffolds constitute a support for osteoblastic adhesion and proliferation, but also microenvironment for stem cells' chemotactism and differentiation [1]. Different sources of living cells are described, as mesenchymal stem cells (MSCs), adipose-tissue derived stem cells, skin derived multipotent stem cells, or oral cavity MSCs, presenting compatible immunophenotype or morphology [2, 3]. The main interest of the use of bone marrow derived stem cells is their osteogenic potential for neoangiogenesis. Several therapeutic applications are developed in the field of bone and cartilage defect treatments, based on the osteoinductive and osteoconductive properties of these materials but also on the intrinsic physiological regenerative properties of bone [3].

Nanofibrous and microporous membranes are very suitable to promote bone regeneration as a mimetic extracellular matrix. By electrospinning, matrices of different synthetic and natural polymers are built, with nanofibers of diameters closest to the size of collagen nanofibers (50 to 500 nm). The electrospun randomized nanofiber network and the created micropores (diameter 100 *μ*m) mimic the pattern of the connective tissue matrix [4, 5]. Thus, nanofibers promote osteoblast adhesion [6], proliferation [7], differentiation [8], and biomineralization [6].* In vivo*, electrospun matrices of poly(*ε*-caprolactone) (PCL) showed favorable results for bone regeneration [9–11].

The functionalization of these matrices enhances the bone regenerative process. To functionalize at a nanoscale level is very convenient. It allows the concentration of many different functions in a small volume and presents the advantage of increasing the quality of targeting while controlling the cost and delivery kinetics of the active molecules [12]. Thus, the strategy of functionalization of nanofibers by nanoreservoirs of BMP-2 or BMP-7 showed a great efficiency for bone regeneration and increased the differentiation of MSCs (mesenchymal stem cells), accelerating the tissue regeneration* in vivo* [9, 10, 13, 14]. These different nanofiber scaffolds with nanoreservoirs are efficient proregenerative biomimicking implants for bone regeneration. The next challenge of these smart active nanomaterials is to be able to promote normalization of implantation site. Indeed, some pathologies or treatments can modify drastically properties of implantation bone site and compromise the regeneration, for example, in contexts as aging or genetical and metabolic skeletal diseases, after tumors resection, severe traumas, and in rare diseases with bone hypotrophy or structural defects.

The skeletal phenotype described in patients with ectodermal dysplasia and in the Tabby (Ta) mutant experimental mouse model is characterized by craniofacial dysmorphia, marked alveolar bone hypotrophy, bone structural defects leading to endosseous implants, and jaw bone grafts postoperative complications. The Ta mutant mouse corresponds to the experimental model of ectodermal dysplasia genodermatosis, with a satisfactory isomorphism, and presents a spontaneous mutation of* Ta* gene exon 1, the mouse homologous of* EDA* gene, mutated in humans affected by ectodermal dysplasia. Therefore, the Ta model was used to evaluate* in vivo* the bone response after microsurgical lesion in the context of ectodermal dysplasia (ED). The phenotypic spectrum of Ta model integrates craniofacial and postcranial bone morphological, structural, and metabolic anomalies [15]. For example, dysplastic zones in the tail vertebrae with histological and structural trabecular bone defects have been observed. Moreover, dental morphotypes with agenesis and morphological defects have been extensively characterized and mimic human phenotype [16]. In our study, only Ta males were used presenting a severe phenotype, in order to avoid any variability linked to genetic or hormonal status. Wild-Type (WT) mice were used as control group. Clinically, the management of maxillary bone defects represents a challenge with indications of extensive bone grafting [17–19]. Despite the fact that autogenous or allogenic bone grafting is considered as a gold standard, some complications were described, especially in the context of genetical diseases, leading to the development of bone tissue-engineering application [20]. The use of biomembrane with nanoreservoirs embedding different dimers like BMP-2 and Ibuprofen is a promising approach to compensate the bone defects linked to the EDA/Ta mutation, combining biomaterials, cells, and signaling molecules, essential in bone bioengineering, osteogenesis, and neoangiogenesis [3]. The role of Ibuprofen is to modulate inflammation in a context of NF-KB pathway dysfunction, this pathway being essential in the inflammation process regulation. On the other hand, BMP-2 promotes bone formation by stimulating osteoblasts differentiation, proliferation, and migration, allowing accelerated bone healing [21]. BMP family molecules are widely used in different homodimeric and heterodimeric associations for the management of bone fractures, skeletal defects, nonunions, or osteonecrosis [22]. The most studied BMP isoforms are BMP-2 and BMP-7, used as recombinant human BMP in the treatment of skeletal diseases, these isoforms playing a major role during bone embryogenesis and postnatal bone homeostasis and remodelling [23]. Nevertheless, clinical use of BMPs isoforms is still controversial, with a limited number of controlled comparative trials, which leads us to study in an experimental mouse model the biological effects of BMP-2 release on maxillary jaw bone neoformation.

The aim of the study is to produce a proregenerative biomimicking implant carrying anti-inflammatory and osteoinductive properties in order to enhance maxillary bone regeneration in a model of ectodermal dysplasia, the Tabby mutant mouse model. Our team focuses on the kinetics of molecules release* in vivo* from PCL functionalized biomembranes, which are crucial in osteogenic differentiation of stem cell control.

## 2. Materials and Methods

### 2.1. Products

Polycaprolactone (PCL; MW 80 KDa) analytical grade and Ibuprofen (50 *μ*g/mL) were purchased from Sigma Aldrich (St. Louis (MO), USA), BMP-2 (200 ng/mL) was purchased from Euromedex (Souffelweyersheim, France), and chitosan (Protasan UPCL 113, 500 *μ*g/mL) was purchased from Novamatrix (Norvège).

### 2.2. Biomembrane

PCL nanofibrous biomembranes were obtained by electrospinning and bifunctionalized using the nanoreservoir technology, producing BMP-2/Ibuprofen nanoreservoirs [11]. The PCL was dissolved in a mix of dichloromethane and dimethylformamide (DCM/DMF 50/50). Electrospinning allowed producing biomembranes of entangled polymer nanofibers. A syringe of 5 mL ejected the solution through a high-voltage electric field (15 kV). The solvent evaporated and the PCL formed fibers are recovered at the collector (20 × 20 cm^2^ aluminium foils). The 40 *μ*m-thick PCL membranes were soaked in 70% ethanol and exposed under ultraviolet light for 30 min to be sterilized. The electrospun fibers were 544 +/− 88 nm in diameter (mean over 50 fibers) as previously described [13].

### 2.3. Buildup of the Nanoreservoirs

For the biological activity experiments, (BMP-2/chitosan)_3_ and (Ibuprofen/chitosan)_3_ were built up on the PCL scaffold. The membrane was washed for 15 min in MES buffer (40 mM, pH 5.5) and then in chitosan (500 *μ*g/mL) before to immerse it again in MES buffer and in the BMP-2 solution (200 ng/mL). Each immersion must last 15 min. The cycle is repeated three times with BMP-2 and three times with Ibuprofen (50 *μ*g/mL). The concentrations of the solutions were taken from the literature and previous study [13]. Chitosan has a positive charge with a p*K*_*a*_ of 6.5. BMP-2 has a positive global charge in this experimental condition (MES pH 5.5) with its isoelectric point of 8.5 while Ibuprofen has a negative global charge (isoelectric point of 4.91). But BMP-2 is an amphoteric protein with negatively charged extremities allowing the layer by layer buildup. The objective was to obtain nanoreservoirs distributed randomly on the surface of PCL nanofibers as shown in previous study [11]. Encapsulated in the nanoreservoirs of chitosan, BMP-2 and Ibuprofen are protected and available for cell activity.

### 2.4. Scanning Electron Microscopy (SEM)

SEM allowed characterizing the morphological structure of nanoreservoirs on the PCL biomembrane as previously described [24] and the morphology of the osteoblasts on the scaffolds after 4 days of culture. The biomembrane was fixed and dehydrated in ethanol baths of increasing concentration (25%, 50%, 75%, 90%, and 100%) each for 10 min. It was placed on a specimen holder and fixed with carbon-conductive adhesive tape. Hexamethyldisilazane (HDMS) was deposited on the sample. The objective was to observe the nanofibrous substructure, the size and the porosity of the fibers, and the distribution and the size of the nanoreservoirs.

### 2.5. Adsorption of Ibuprofen on PCL Membrane

To quantify the Ibuprofen attached to the biomaterials, we recovered the soaking solutions after each adsorption cycle. The optical density was measured at 200 and 350 nm. The amount of Ibuprofen was then determined using a standard curve (Supporting 1).

### 2.6. *In Vitro* Characterization

Human primary osteoblasts (Hob) (PromoCell GmbH, Heidelberg, Germany) were grown in a “Specific Medium” with “Supplement Mix” (PromoCell GmbH, Heidelberg, Germany). The cells were incubated at 37°C in a humidified atmosphere of 5% CO_2_. When cells reached subconfluence, they were harvested with trypsin and subcultured on nonfunctionalized PCL or Ibu, BMP2, or BMP2/Ibu functionalized PCL membranes in 24-well plates. The membranes were treated with 70% ethanol and sterilized by 30 min exposure to UV light before cell seeding. For this, the membrane was punched to the well size and locked in. The cell viability and proliferation were measured by the AlamarBlue® test (Fisher Scientific, Illkirch, France). Before AlamarBlue analysis, samples have been moved in a new well in order to measure only the metabolic activity of cells attached on the scaffold. The osteoblasts were also studied by immunofluorescence for the expression of osteopontin and BSPII after 14 days of culture. Briefly osteoblasts were fixed with 4% paraformaldehyde (PFA) for 10 min at 4°C, saturated with 0.1% Triton X-100 and 1% BSA for 1 h, and then rinsed three times with PBS. Primary antibodies were incubated overnight at 4°C at 1/200: rabbit anti-BSPII (sc-73497, Santa Cruz Biotechnology, Clinisciences, Nanterre, France) and mouse antiosteopontin (OPN, sc-10591, Santa Cruz Biotechnology, Clinisciences, Nanterre, France). After three washings with PBS, samples were incubated for 1 h with anti-rabbit Alexa 488 or anti-mouse Alexa 488 (Molecular Probes, Fisher Scientific, Illkirch, France) and then with Alexa Fluor 594 Phalloidin (1/200, Molecular Probes, Fisher Scientific, Illkirch, France) for 10 min and 5 min with 200 nM 4′,6-diamidino-2-phenylindole (DAPI, Euromedex, Souffelweyersheim, France). The samples were observed under an epifluorescence microscope (Olympus DP73).

### 2.7. *In Vivo* Microsurgical Protocol

The experimental protocol fulfilled the authorization of the “Ministère de l'Enseignement Supérieur et de la Recherche” under the agreement number 01716.02. The Ethics Committee of Strasbourg named “Comité Régional d'Ethique en Matière d'Expérimentation Animale de Strasbourg (CREMEAS)” specifically approved this study. Under general anesthesia, a maxillary bone lesion was created in the diastemal area with a dental bur (500 *μ*m) after gingival incision (Supporting 2A, B). On one side, bifunctionalized BMP-2/Ibuprofen scaffold or functionalized Ibuprofen scaffold or functionalized BMP-2 scaffold was implanted while the other side served as a control with the same lesion but without scaffold or with nonfunctionalized membrane (Supporting 2C). The mucosa was closed with biological glue (3M Vetbond™ Tissue Adhesive, Fisher Scientific, Illkirch, France) (Supporting 2D).

### 2.8. *In Vivo* Microcomputed Tomography (Micro-CT) Analyses

To study the evolution of bone response, we conducted a longitudinal postoperative follow-up using microcomputed tomography. The X-ray microtomography acquisitions were performed under general anesthesia after 7, 21, and 30 days. A spatial isotropic resolution of 50 *μ*m was used for the acquisitions. Volumic analyses of bone lesions followed the definition of a cubic region of interest (ROI) framing these lesions.

### 2.9. Histological Analyses

Osteoblasts cultured for 4 days on PCL biomembranes were fixed for 10 min with 4% paraformaldehyde and stained with hematoxylin-eosin. The histological analyses of neoformed bone structures in WT and Ta mutant mice were conducted at 30 days postoperative. Maxillaries were fixed for 24 h with 4% paraformaldehyde, decalcified in EDTA 15% at 37°C for one week, and embedded in paraffin. Serial sections (10 *μ*m) were stained with hematoxylin-eosin. Sections were observed on a Leica DM4000B microscope.

### 2.10. Statistical Analyses

Statistical analyses were performed using Student's* t*-test. Statistical significance was evaluated by one-way ANOVA (SigmaStat, Jandel GmbH, Erkrath, Germany). All data were expressed as mean ± standard deviation (SD). *p* < 0.05 was considered as statistically significant.

## 3. Results 

### 3.1. Characterization of the Scaffolds

**Figure 1:**
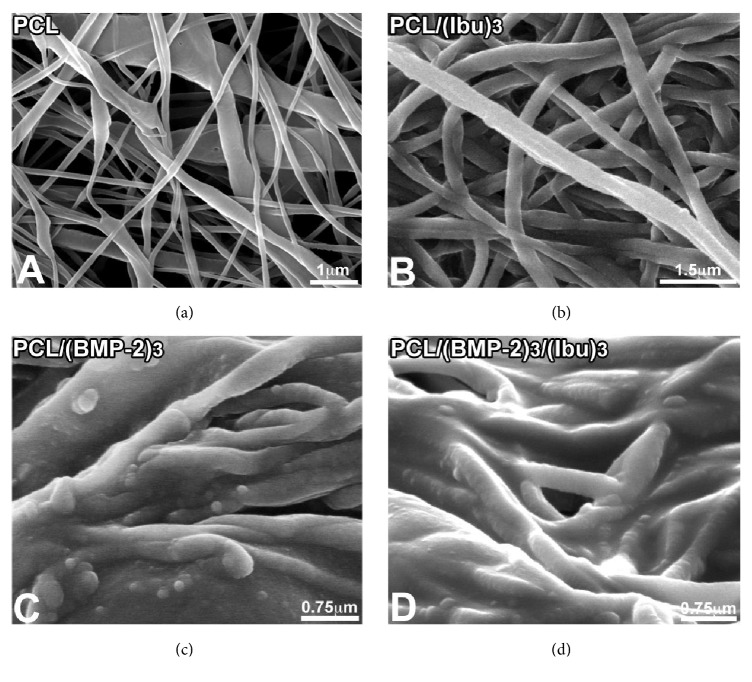
Scanning electron microscopy (SEM) observations of nonfunctionalized PCL scaffolds consisting of nonwoven electrospun nanofibers (a), PCL functionalized with Ibuprofen (PCL/(Ibu)_3_, 50 *μ*g/mL) (b), BMP-2 (PCL/(BMP-2)_3_, 200 ng/mL) (c), and BMP-2/Ibuprofen (PCL/(BMP-2)_3_/(Ibu)_3_) nanoreservoirs (d).

The polycaprolactone fibers and nanoreservoirs were characterized by scanning electron microscopy (SEM) (Figure 1). Their distribution was random. The PCL scaffolds (Figure 1(a)) evidenced a nonwoven mesh like structure with a large surface area per volume ratio. The electrospun fibers were 544 +/− 88 nm in diameter, porosity corresponding to interfibers spaces ranged between 400 nm and 2 *μ*m. The nanofiber diameter of the developed PCL nanofibers falls within the range of the native collagen nanofibers diameter present in extracellular matrix (ECM). The nanoreservoir technique was used to decorate the surface of the nanofibers with BMP-2/Ibuprofen. Figures 1(c) and 1(d) showed BMP-2 nanoreservoirs tightly grafted on the surface of the electrospun nanofibers. Ibuprofen was homogeneously distributed along the PCL fibers (Figures 1(b) and 1(d)). As previously described [13], the amount of BMP-2 incorporated into the nanoreservoirs was 0.73 *μ*g/cm^2^ by using Quartz crystal microbalance with dissipation monitoring (QCM-D).

The recovery of the dipping solutions for Ibuprofen during the functionalization of the scaffold allowed measuring the fixed amount of Ibuprofen. The amount of Ibuprofen was constant for the first two cycles (Table 1). In the third cycle a small amount of Ibuprofen was fixed, that is why we have not done more cycles. The spectrophotometry did not show any passive release of Ibuprofen (not shown).

### 3.2. Biocompatibility of the scaffolds

**Figure 2:**
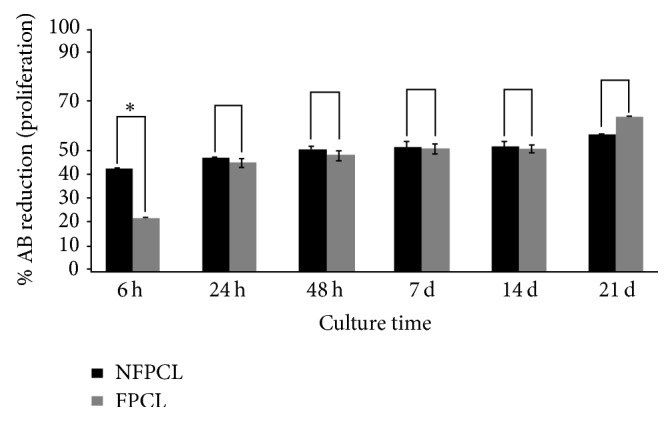
*In vitro* proliferation of human primary osteoblasts (Hob) on nonfunctionalized (NFPCL) and BMP-2/Ibu bifunctionalized (FPCL) scaffolds. The error bars represent the standard deviation. ^*∗*^*p* < 0.1.

**Figure 3:**
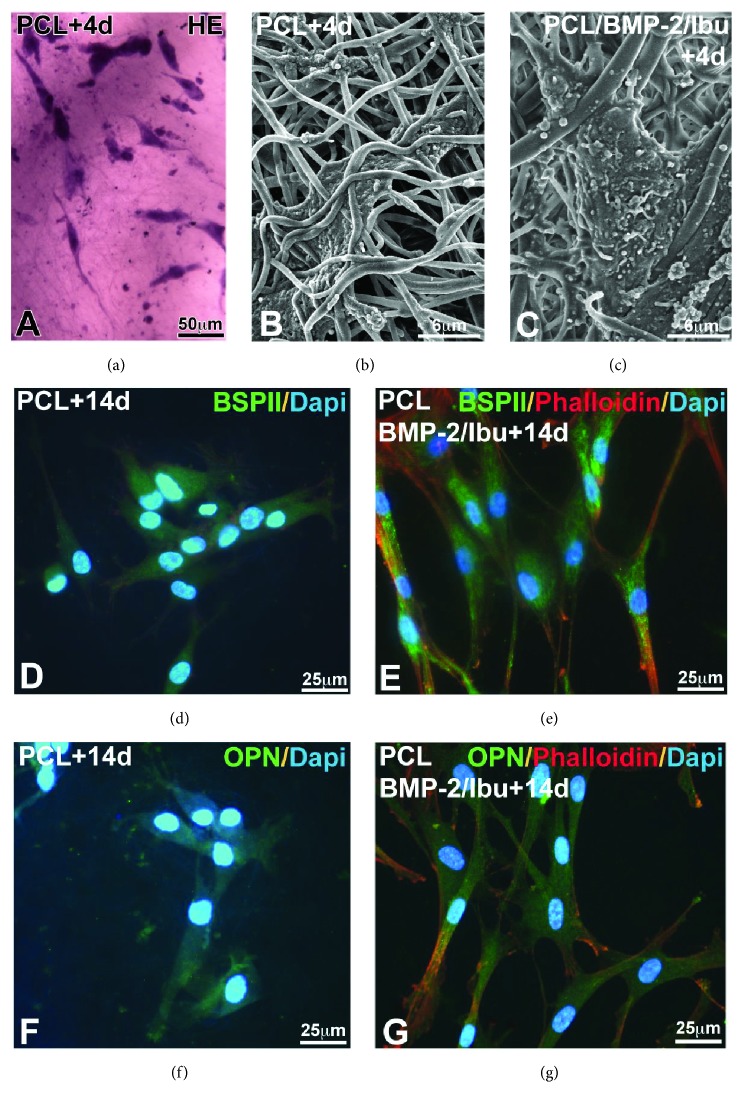
Morphology of Hob using hematoxylin-eosin (HE) staining (a) and SEM (b, c) after 4 days of culture and expression of BSPII (d, e) and osteopontin (f, g) in human primary osteoblasts after 14 days culture on PCL scaffold (d, f) and on BMP-2/Ibu bifunctionalized scaffold (e, g). Cells are well spread and migrate along nanofibers (a–c). The nuclei were labeled with DAPI and actin with phalloidin. BMP-2: bone morphogenic protein 2, BSPII: bone sialoprotein 2, Ibu: Ibuprofen, and Osp: osteopontin.

The nanofibrous structure enables cell migration and growth as well as nutrient and bioactive molecule diffusion. Biocompatibility evaluation of the PCL scaffolds was based on human primary osteoblasts (Hob) metabolic activities analyses using AlamarBlue test (Figure 2). Cell morphology, adhesion on the PCL scaffolds, and expression of osteopontin and BSPII, which are noncollagen bone matrix proteins, were evaluated (Figure 3).

The cells were seeded on the scaffolds and then cultured for 21 days in the osteoblastic medium. The AlamarBlue reduction percentage, followed over times 6, 24, and 48 h and 7, 14, and 21 days of culture, confirmed the viability of the cells on both types of scaffolds (without or bifunctionalized with BMP-2/Ibu). The results showed that, after 6 h of culture, the osteoblasts had a higher metabolic activity on the uncoated scaffolds. This activity increased after 24 h and was identical on both types of scaffolds. This activity was constant up to 14 days and increased again after 21 days of culture. The metabolic activity of osteoblasts is significantly more important on bifunctionalized scaffolds than on control scaffolds. The bifunctionalized scaffolds were therefore not toxic for the osteoblasts and can be used in* in vivo* implantation experiments.

After 4 days of culture, the Hob was observed by hematoxylin-eosin (HE) staining (Figure 3(a)) and by SEM (Figures 3(b) and 3(c)). The cellular morphology of osteoblasts was identical after 4 days of culture on nonfunctionalized membrane and on bifunctionalized membrane. The cells were spread on the membrane surface and inside. The numerous cellular extensions infiltrated between the nanofibers showing a satisfactory biocompatibility of the biomembrane (Figures 3(b) and 3(c)).

After 14 days of culture, the Hob was tested for their expression of bone specific proteins: bone sialoprotein 2 (BSPII) and osteopontin (OPN). The immunofluorescence images showed that osteogenesis occurred successfully in both types of scaffolds: PCL (Figures 3(d) and 3(f)) and PCL/BMP-2/Ibu (Figures 3(e) and 3(g)). However, the qualitative enhancement of protein expression by the bifunctionalization is significant* in vitro*, even after 14 days (Figures 3(e) and 3(g)).

### 3.3. Effects of the Bifunctionalized Scaffold on the Bone Neoformation in WT and Ta Mice by Micro-CT

**Figure 4:**
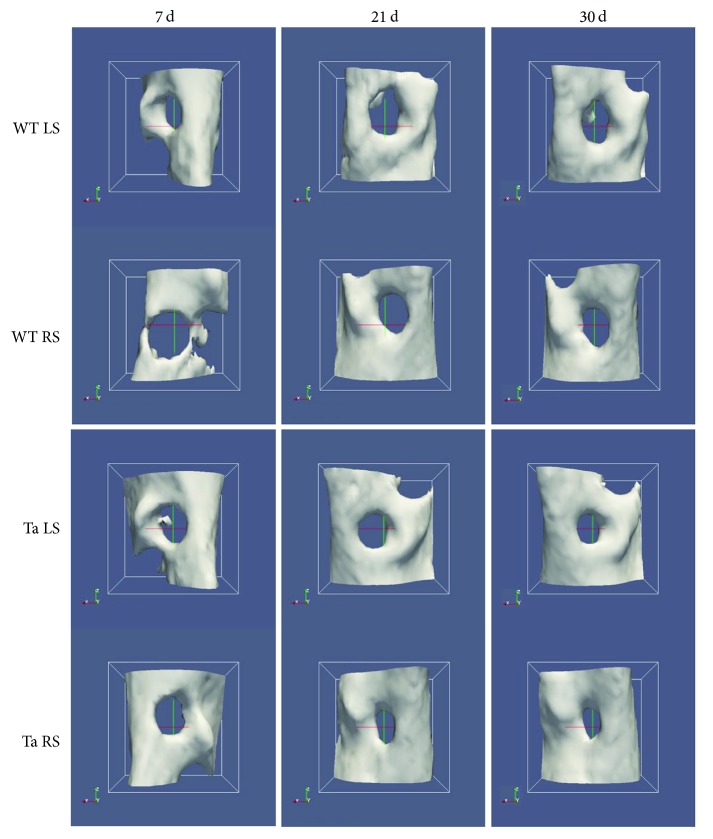
Micro-CT tridimensional reconstructions of bone lesions in WT and Ta mice illustrating the bone closure between day 0 and day 30. WT control side (LS) and WT treated side (RS); Ta control side (LS) and Ta treated side (RS).

**Figure 5:**
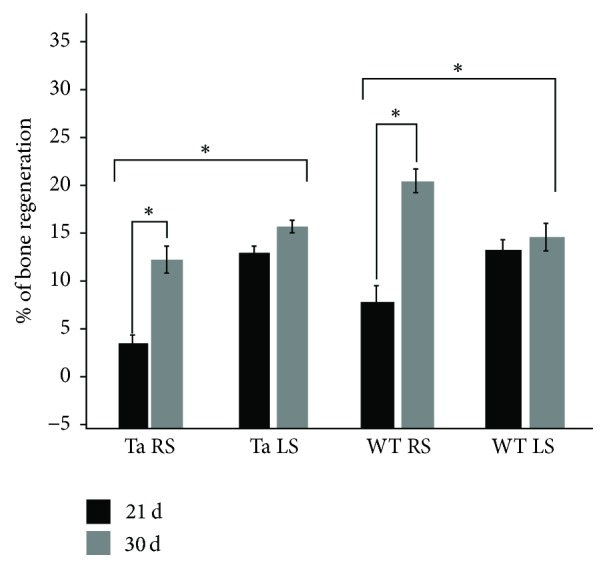
Differential effects of the BMP-2/Ibuprofen bifunctionalized scaffold on bone regeneration in Wild-Type (WT) and Tabby (Ta) mice after 21 and 30 days of implantation. The bone regeneration was evaluated as a differential in the bone lesion volume determined by micro-CT between T0 and 21 and 30 days in the presence or absence of the biomembrane. LS: control scaffold and RS: BMP-2/Ibu scaffold. The error bars represent the standard deviation. ^*∗*^*p* < 0.1.

Effects of the BMP-2/Ibuprofen bifunctionalized PCL scaffold on maxillary bone regeneration were evaluated using micro-CT analyses. In the WT mice, no difference at day 21 was observed between the treated side (RS) and the control side (LS). Positive effect of the scaffold was observed in WT on the treated side (+13%) between day 21 and day 30 (Figure 4, WT RS), which was not observed on the control side (Figure 4, WT LS). An average bone neoformation of 14.4% at 30 days for the control lesions was observed, compared to 21% for the lesions treated with BMP-2/Ibu scaffold (*p* < 0.05) (Figure 5).

In the Ta mice, the treatment with BMP-2/Ibu scaffold leaded to a lower bone neoformation than control lesions at day 21 (4% versus 13%). We observed a positive effect on the treated side between day 21 and day 30 (+8%), which was not observed on the control side (Figures 4 and 5).

### 3.4. Histological Analyses of the Effects of Different Scaffolds on the Bone Neoformation in WT Mice

**Figure 6:**
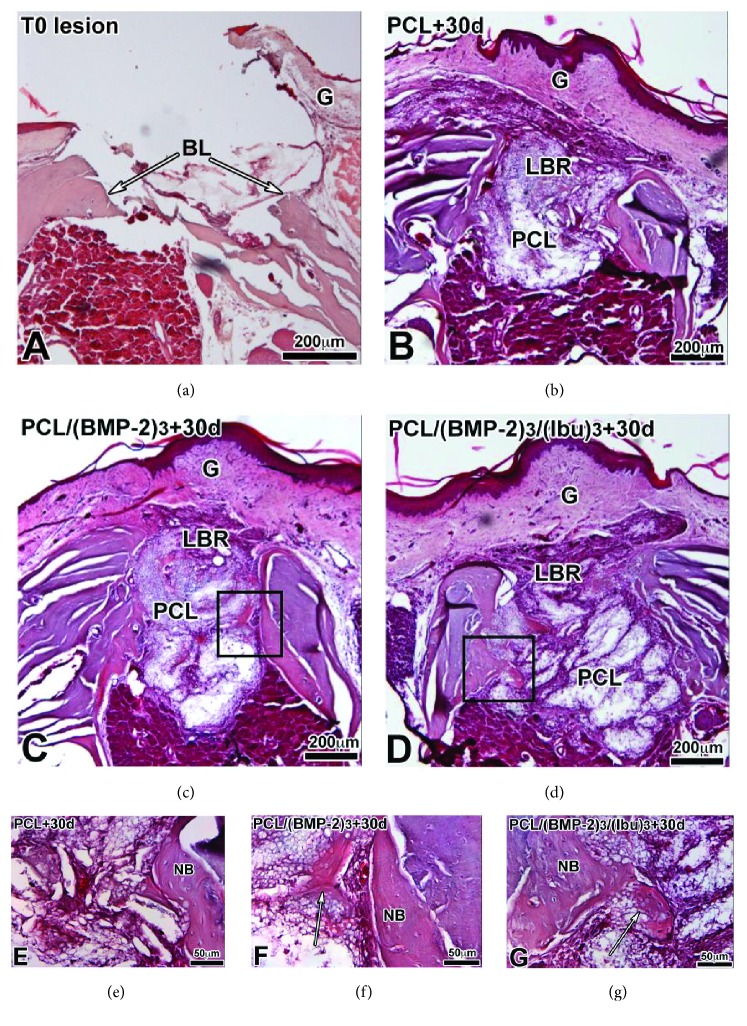
Histological sections of the lesion at T0 (a) and after 30 days implantation of PCL (b, e), PCL/(BMP-2)_3_ (c, f), and (PCL/(BMP-2)_3_/(Ibu)_3_) (d, g) scaffolds stained with hematoxylin-eosin. After 30 days the gingiva is healed and bone regenerates on both sides of the lesion with the 3 different scaffolds. When the membrane is functionalized with BMP-2 or BMP2/Ibu, we also observe bone regeneration inside the membrane (arrows in (f) and (g)). The osteoblasts migrate into the membrane. BL: bone lesion, G: gingiva, LBR: lesion with bone regeneration, NB: neoformed bone, and PCL: scaffold.

We first compared neoformed bone after PCL, PCL/BMP-2, and PCL/BMP-2/Ibu implantation for 30 days (Figure 6) on paraffin sections stained with hematoxylin-eosin that allows visualization of the extracellular bone matrix and collagen type I. After 30 days, the gingiva was healed and bone regenerated on both sides of the lesion with the 3 different scaffolds (Figures 6(b)–6(d)). When the membrane was functionalized with BMP-2 or BMP-2/Ibu, we also observed bone regeneration inside the membrane (Figures 6(f) and 6(g), arrows). We did not observe any noticeable difference in bone regeneration between BMP-2 and BMP-2/Ibu scaffolds.

### 3.5. Histological Analyses of the Effects of the Bifunctionalized Scaffold on the Bone Neoformation in WT and Ta Mice

**Figure 7:**
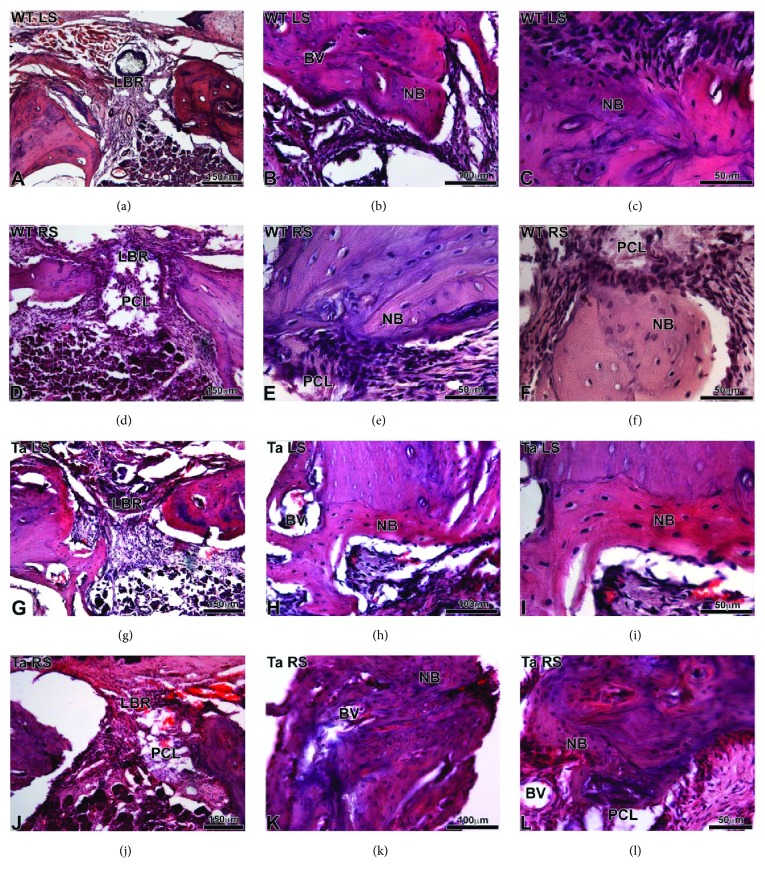
Histological sections of WT (a–f) and Ta (g–l) maxillary bone stained with hematoxylin-eosin after 30 days of implantation. (a–c) and (g–i) corresponded to the lesion on the left side without scaffold. (d–f) and (j–l) corresponded to the lesion on the right side with bifunctionalized BMP-2/Ibuprofen scaffold. BV: blood vessel, LBR: lesion with bone regeneration, NB: neoformed bone, and PCL: bifunctionalized scaffold.

Histological analyses, at 30 days postoperative (Figure 7), confirmed the micro-CT analyses and showed neoformed bone (NB) with regular structure at the level of lesions treated by BMP-2/Ibu scaffold (Figures 7(d)–7(f), 7(j)–7(l)) in WT and Ta mice. Neovascularization was more important at the level of the lesion treated with PCL/BMP-2/Ibu scaffold especially for the Ta mice (Figure 7(j)).

## 4. Discussion

### 4.1. Effects of the Functionalized Biomembrane in WT and Mutant Ta Mice

Polycaprolactone (PCL) membrane revealed an osteoconductive effects and BMP-2 stimulated the bone production by its osteoinductive properties [21, 25]. Functionalization with BMP-2 was already described in previous study and approved by American authorities (FDA). In our study, we adopted a system consisting in direct LbL-based nanoimmobilization of BMP-2, allowing protection of the growth factor and the use of lower concentrations (three adsorptions steps with a 200 ng/mL solution) compared to the soaking approach. Ibuprofen appears to stimulate neovascularization according to other studies, this effect being based on an increased secretion of VEGF and endothelial cell proliferation [26]. The bioavailability of the Ibuprofen entrapped in the nanoreservoirs is improved, compared to scaffolds electrospun with Ibuprofen in solution [27, 28]. Moreover, use of lower concentrations allows a reduction of cell toxicity and genotoxicity, the last one being observed on mouse bone marrow cells in contact with Ibuprofen [29].

The maxillary bone regeneration based on nanoreservoirs functionalized PCL biomembranes showed promising results in WT mice; nevertheless the use of Ibuprofen inhibited early bone neoformation in mutant Ta mice. In the Ta and WT mice, the increase in bone formation peaks between the 21st and the 30th days following the surgery (*p* < 0.05). This can be explained by the diffusion kinetic of the Ibuprofen entrapped in the nanoreservoirs. Despite the absence of significative effect of the bifunctionalized membrane in the Ta mutant mice at 30 days, a more important stimulation of osteogenesis is observed between the 21st and 30th day in the treated lesion compared to control lesion. Only BMP-2 and BMP-2/Ibuprofen membranes were used on the protocol indeed; based on our previous experimental results [11, 13] and literature data [30], we assumed the absence of positive biological effects on osteoblast proliferation of an Ibuprofen functionalized membrane, in the absence of an osteoinductive molecule as BMP-2.

The altered effects of the biomembrane in the Ta mice could be potentially linked to the bone metabolic and structural anomalies associated with the mutation [18, 31]. These genetically determined bone abnormalities associated with the* Ta* mutation could not be integrally compensated by the biological effects of the bifunctionalized PCL biomembrane. We assume the absence of early effects in the Ta model linked to bone physiopathology and a negative compensation of BMP-2 effects by the mutation.

### 4.2. Potential Development of the Model

The main interest of this mouse model consists in the possibility of evaluating bone response in the context of* EDA/Ta* mutation. Besides the characterization of dental and skeletal phenotypes linked to HED [32, 33], this mouse model allowed a dynamic approach of bone response kinetic, based mainly on* in vivo* micro-CT and histological techniques. More accurate micro-CT approaches, with higher isotropic resolution, will be developed, based on the use of synchrotron micro-CT techniques or nano-CT. This high-resolution micro-CT will allow deep ultrastructural phenotyping of the neoformed bone and its differences between Wild-Type and different genetically modified mice. These micro-CT acquisitions will lead to tridimensional morphometric characterizations of the native and neoformed bone, with description of parameters like trabecular bone volume, trabecular number, thickness, or intertrabecular spaces. The soft tissues ingrowth and morphological modifications of the scaffold will also be studied by synchrotron micro-CT. The vascular ingrowth process, important for postoperative bone regeneration, will also be studied on this model based on K-edge subtraction micro-CT using synchrotron lights [34]. The development of this model is essential both to understand the physiopathological mechanisms and in preclinical research applied to genetical rare diseases. Furthermore, this mutant mouse model makes experimental approaches of bone grafting and osteointegration complication mechanisms in patients affected by HED possible. The surgical protocol applied in Ta mice allowed the exploration of altered jaw bone response and the potential osteogenic effects of PCL biomembranes. The characterization of bone response in Ta mice can be adapted to other mutant mice presenting skeletal abnormalities and described in the literature like the* Lrp4* mutated sclerosteosis mouse model [35] or the mutant FKBP51^V55L^ for Paget's disease [36]. Indeed, applications of this microsurgical protocol to other mouse models of genetic diseases with skeletal defects will allow the* in vivo* study of the maxillary bone response in different pathological contexts. Beyond the maxillary location, it will be possible to analyze the bone response of Ta mice in other anatomical locations, as calvaria or long bones. Significative osteoinduction using BMP-2 was demonstrated in other animal models in calvaria like rabbit or mice [37, 38].

### 4.3. Potential Development of the Biomembrane

The design of the scaffold, the components, and the functionalization with different signaling molecules can be modified [39] and adapted according to the pathological context, the genetical defect, or the anatomical site [29]. The membrane may be formed from different materials with specific properties of biocompatibility, cytotoxicity, resorbability, or osteogenic capacity. Different polymers are available, as PCL, PCL associated with other materials as PLA, or other electrospun substances like polystyrene [39, 40]. The size and the morphology of the nanofibers are the main parameters that can be controlled, by modulating for example the flow rate or the polymer concentration [5]. The thickness, the microstructure, and the porosity of the scaffold are the other controlled parameters of the biomembrane. Interconnected porosity is a crucial factor to obtain sufficient neovascularization [41].

There are many perspectives and potential therapeutic clinical applications, like functionalization with mesenchymal stem cells or osteoblasts, use of different BMP isoforms homodimeric and heterodimeric associations [9, 10], or different molecules like statins or hypoxia-mimetic agents [42]. Experimental use of these molecules was already reported, but with other types of scaffolds like hydrogels [43] or in gelatin nanofibrous scaffold [41]. It might be interesting to combine these substances with the functionalized scaffold by the nanoreservoirs technology. The quantity of nanoreservoirs can be modified by increasing the number of functionalization cycles and thus allowing a longer effect over time.

## 5. Conclusion

Biomembrane-based engineering appears as a promising approach allowing bone regeneration and opens the possibility of developing biomaterials functionalized with different molecules or stem cells. In this study, the association between Ibuprofen and BMP-2 on a PCL membrane makes it possible to have both osteoinductive and anti-inflammatory effects. In the WT mice, the bifunctionalized scaffold showed only a late biological effect, with bone neoformation being observed between day 21 and day 30, whereas in Ta mice, the bone neoformation is lower than control lesions at day 21 and then increased secondarily. We assume that the difference between Ta and WT is linked to bone metabolic alterations. The main research perspectives are to adapt biomembranes to the physiopathology of rare diseases like HED, skeletal dysplasia, or bone tumors and metastases. The combination with other materials, stem cells, and molecules may be benefit to induce bone regeneration. The purposes are to promote cell adhesion, osteogenic differentiation, improve bone formation, and mechanical properties allowing a decrease of postoperative complications prevalence.

## Figures and Tables

**Table 1:** Quantity of adsorbed Ibuprofen on the scaffold per number of cycles.

	First cycle	Second cycle	Third cycle
Quantity adsorbed Ibu 50 *μ*g/ml	24 *μ*g	20 *μ*g	2 *μ*g
Quantity adsorbed Ibu 50 *μ*g/ml on BMP2	30 *μ*g	30 *μ*g	2 *μ*g

## Supplementary Materials

Supplementary 1 (A) Increasing Ibuprofen spectrum in the MES buffer (pH 5.5) and (B) calibration curve of Ibuprofen at 265 nm. The preparation of different concentrations of Ibuprofen for calibration was performed in a MES buffer solution. The range of increasing concentrations was the following: 0.05, 0.1, 0.2, 0.4, 0.6, 0.8, and 1 mg/ml. The solutions were prepared in triplicate. The measurement of the absorbance was carried out between 200 and 350 nm. This calibration gives us a signature of Ibuprofen and useful spectra for quantification.

Supplementary 2 Different stages of the microsurgery: (A) incision of the gingiva, (B) maxillary bone lesion obtained with a dental bur (500 *μ*m), (C) implantation of the biomembrane, and (D) closing of the gingiva with biological glue.
