# Supplementary figures and images for: Maxillary Bone Regeneration Based on Nanoreservoirs Functionalized ε-Polycaprolactone Biomembranes in a Mouse Model of Jaw Bone Lesion
Source: Biomed Res Int. 2018 Feb 26;2018:7380389. doi: 10.1155/2018/7380389 (PMC5846386; doi:10.1155/2018/7380389)

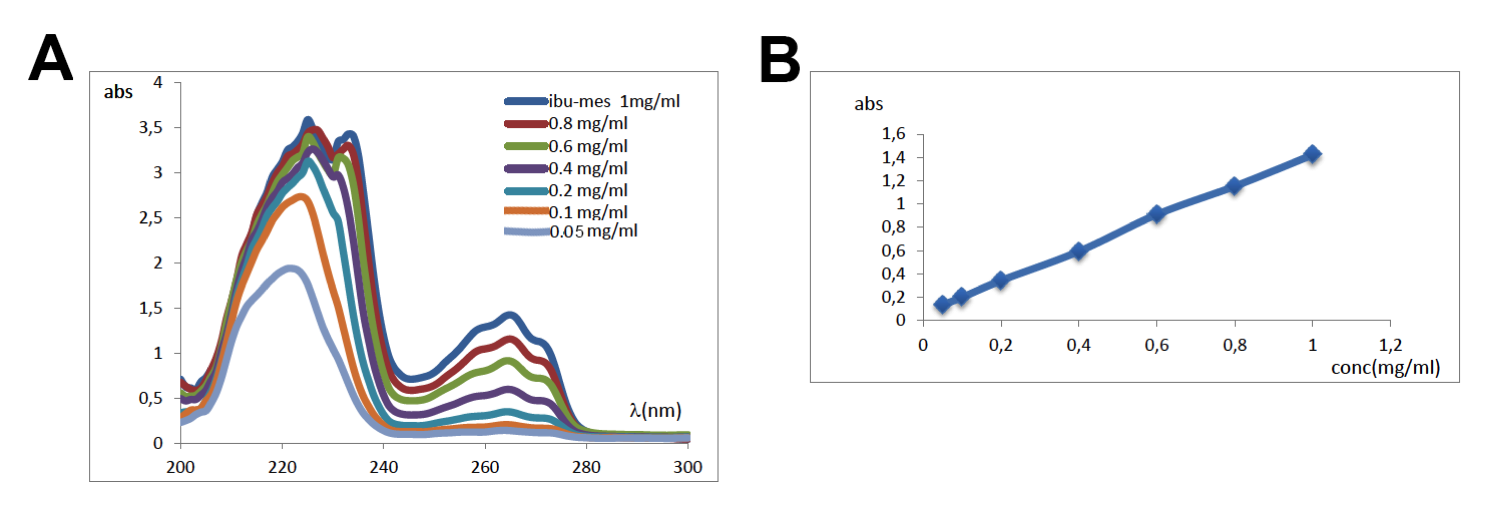

Supplement: Supplementary 1 — (A) Increasing Ibuprofen spectrum in the MES buffer (pH 5.5) and (B) calibration curve of Ibuprofen at 265 nm. The preparation of different concentrations of Ibuprofen for calibration was performed in a MES buffer solution. The range of increasing concentrations was the following: 0.05, 0.1, 0.2, 0.4, 0.6, 0.8, and 1 mg/ml. The solutions were prepared in triplicate. The measurement of the absorbance was carried out between 200 and 350 nm. This calibration gives us a signature of Ibuprofen and useful spectra for quantification. [file 7380389.f1.docx]

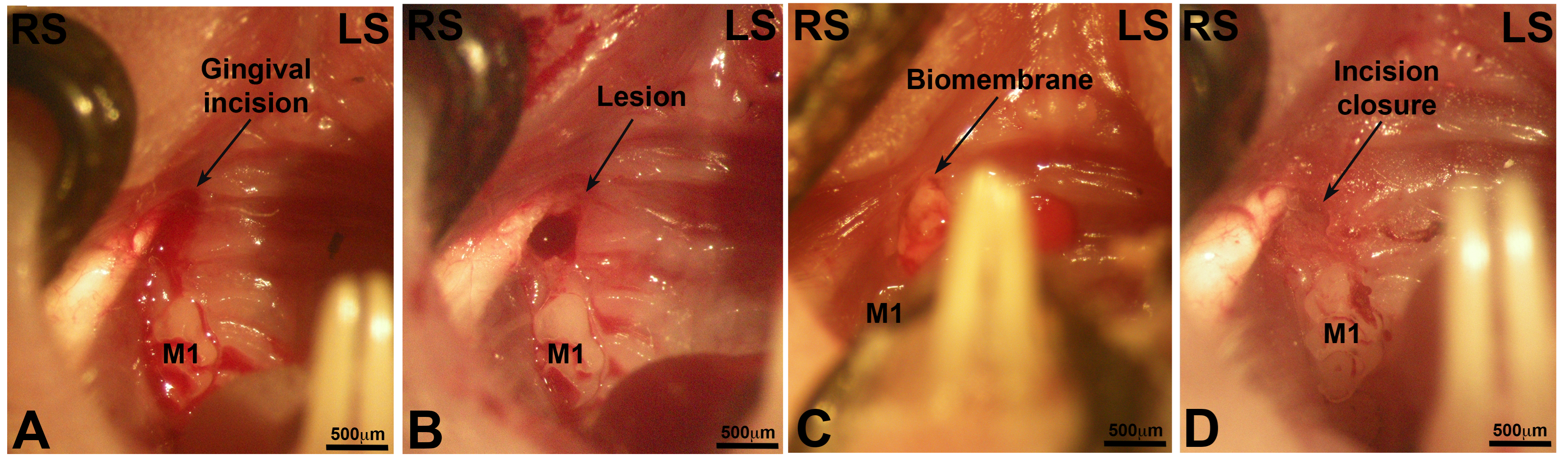

Supplement: Supplementary 2 — Different stages of the microsurgery: (A) incision of the gingiva, (B) maxillary bone lesion obtained with a dental bur (500 μm), (C) implantation of the biomembrane, and (D) closing of the gingiva with biological glue. [file 7380389.f2.docx]
